# Comparison of Liposomal Bupivacaine versus Bupivacaine in Superficial Parasternal Intercostal Plane Block for Cardiac Surgery with Median Sternotomy

**DOI:** 10.5761/atcs.oa.25-00008

**Published:** 2025-04-04

**Authors:** Rong-En Qiu, Yun-Ping Lan, Shan Liu, Xiang-Yu Fang, Yun-Feng Zhang

**Affiliations:** Anesthesiology Department, Quzhou Affiliated Hospital of Wenzhou Medical University, Quzhou People’ s Hospital, Quzhou, Zhejiang, China

**Keywords:** bupivacaine, coronary artery bypass, pain, postoperative, analgesics

## Abstract

**Purpose:** This study aimed to compare the efficacy and safety of liposomal bupivacaine (Lip-BPVC) versus standard bupivacaine (BPVC) for superficial parasternal intercostal plane block in patients undergoing elective coronary artery bypass grafting (CABG) via median sternotomy.

**Methods:** A total of 82 adult patients were randomly assigned to the BPVC group (n = 41) or the Lip-BPVC group (n = 41).

**Results:** The Lip-BPVC group demonstrated significantly lower pain scores at all postinjection time points compared to the BPVC group with fewer opioid analgesics. Lip-BPVC demonstrated an initial heightened inflammatory response postoperatively compared to standard BPVC, indicated by significantly lower levels of pro-inflammatory markers at 24 and 48 hours postinjection with BPVC. However, by 72 hours, inflammatory markers did not differ significantly between Lip-BPVC and BPVC groups. No significant differences were observed between the groups in terms of surgery duration, extubation time, intensive care unit and hospital length of stay, or incidence of postoperative nausea and vomiting.

**Conclusions:** Lip-BPVC initially increased inflammatory markers postoperatively, but levels were comparable to BPVC by 72 hours. It provided superior pain control and reduced opioid use compared to standard BPVC in CABG patients, with similar safety and recovery outcomes.

## Introduction

Every year, over 1 million people worldwide undergo heart surgery via sternotomy, frequently experiencing postoperative pain, especially within the first 48 hours.^1)^ Consequently, effective pain management following cardiac surgery is crucial for patient recovery, reducing complications, and improving overall outcomes.^2)^ Coronary artery bypass grafting (CABG) performed through median sternotomy can hinder recovery and increase the risk of complications such as pneumonia, deep vein thrombosis, and prolonged hospital stays.^3)^ Furthermore, a substantial majority of these surgical patients experience acute postoperative pain: over 80% report its occurrence, 75% describe it as moderate to severe, and fewer than half achieve adequate relief.^4)^ This underscores the critical importance of effective pain management in improving postsurgical outcomes. To address this issue, a multimodal analgesic strategy aiming at sparing opioid use includes the application of peripheral nerve blocks. Among the various techniques in cardiac surgery, fascial plane blocks, particularly the superficial parasternal intercostal plane block (SPIB), have shown the most promise.^1)^ SPIB has emerged as an effective technique for managing postoperative pain in patients undergoing median sternotomy. Notably, it is associated with reduced intraoperative opioid use and shorter hospital stays.^5,6)^

Bupivacaine (BPVC), a long-acting local anesthetic, is commonly used in SPIB to provide effective analgesia.^7,8)^ However, recent advancements have introduced liposomal BPVC (Lip-BPVC), which was approved by the Food and Drug Administration in 2011 for single-dose infiltration or interscalene brachial plexus block, extending the analgesic duration to 24–96 hours.^4)^ Studies have explored its efficacy in various surgeries, such as total shoulder arthroplasty,^9)^ open posterior lumbar laminectomy and fusion surgery with instrumentation,^10)^ lung resection,^11)^ breast reconstruction,^12)^ and colorectal surgery.^13)^ A recent study compared Lip-BPVC with BPVC for intercostal nerve blockade in minimally invasive thoracic surgery lobectomy,^14)^ finding no significant differences in opioid consumption or pain scores postoperatively with both agents under multimodal analgesia. However, another study demonstrated that Lip-BPVC provided longer-lasting analgesia compared to BPVC alone in an equine model of forelimb lameness induced by sole pressure, suggesting Lip-BPVC’s potential superiority for pain management in horses.^15)^ A meta-analysis found that Lip-BPVC resulted in slightly lower postoperative pain scores and reduced opioid consumption compared to BPVC, although the clinical significance of these findings was minimal.^16)^

Despite the potential advantages of Lip-BPVC, there is a paucity of robust clinical data directly comparing its efficacy with that of standard BPVC in the context of cardiac surgery. This prospective, randomized controlled trial aims to compare the efficacy of Lip-BPVC versus standard BPVC in SPIB for patients undergoing elective CABG. The study would evaluate postoperative pain scores, opioid consumption, inflammatory response, and recovery outcomes. By determining whether Lip-BPVC offers superior pain control and better overall outcomes compared to conventional BPVC, this research seeks to enhance pain management strategies in cardiac surgery, ultimately improving patient recovery and reducing complications.

## Materials and Methods

### Participants

**Fig. 1:**
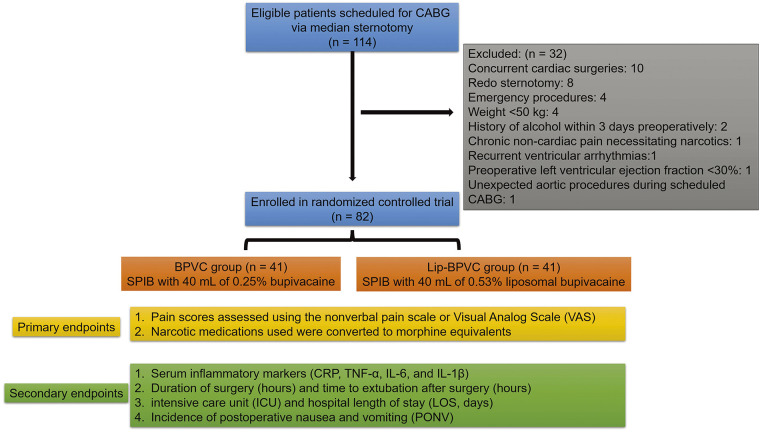
Study flow diagram demonstrating eligibility, exclusions, and analysis. CABG: coronary artery bypass grafting; Lip-BPVC: liposomal bupivacaine; SPIB: superficial parasternal intercostal plane block; CRP: C-reactive protein; TNF-α: tumor necrosis factor-alpha; IL: interleukin.

The study included adult patients (age ≥18 years) scheduled for elective CABG between May 2020 and May 2024, all of whom underwent open sternotomy with saphenous vein graft removal using an open incision technique. Exclusion criteria included concurrent cardiac surgeries, redo sternotomy, or emergency procedures. Additional exclusions were as follows: weight <50 kg; pregnancy or lactation; history of alcohol, narcotic, or illicit drug abuse within 3 days preoperatively; perioperative steroid use requiring narcotic analgesics; chronic noncardiac pain necessitating narcotics; significant liver or kidney disease; allergy to local anesthetics; recurrent ventricular arrhythmias; a preoperative left ventricular ejection fraction (LVEF) <30%; existing pulmonary diseases (such as pulmonary fibrosis or severe chronic obstructive pulmonary disease); neurological disorders; and unexpected valve or aortic procedures during scheduled CABG. Ultimately, a total of 82 patients were enrolled in the study, with patients randomized into BPVC and Lip-BPVC groups using a computer-generated random number table, with 41 patients in each group (**Fig. 1**). This study was approved by the Ethics Committee of our hospital, and all patients provided informed consent before participating.

### Intraoperative management

During intraoperative management, a standardized perioperative analgesic regimen was implemented for both groups of patients undergoing general anesthesia with endotracheal intubation. Anesthesia induction included etomidate and succinylcholine, followed by maintenance using continuous infusions of sufentanil and propofol, administered via target-controlled infusion pumps. SPIB from ribs 2 to 6 were performed by a single cardiothoracic surgical physician assistant under blinded conditions, with medications drawn from concealed vials into sheathed sterile syringes. Participants, the cardiothoracic team, anesthesiologists, and nursing staff were blinded to group allocation. Patients in the BPVC group received 40 mL of 0.25% BPVC for SPIB, while those in the Lip-BPVC group received 40 mL of 0.53% Lip-BPVC for SPIB. All SPIB procedures were performed prior to complete closure of the chest in the operating room. Postoperatively, patients received fentanyl boluses until transition to patient-controlled analgesia (PCA), which included fentanyl or hydromorphone for patients with an allergy or intolerance to fentanyl. PCA was administered for the initial 48 hours postoperatively with predefined settings for loading dose, bolus dose, lockout interval, and maximum hourly limit. Following discontinuation of PCA, patients were managed with oral analgesics such as oxycodone–acetaminophen or hydrocodone–acetaminophen, with rescue doses of ketorolac or tramadol available if needed for inadequate pain relief.

### Outcome measurements

The time of administration of the parasternal nerve block was designated as Time 0. Outcome measurements included primary endpoints of pain scores ranging from 0 to 10, assessed using the nonverbal pain scale or visual analog scale at 8, 12, 24, 48, and 72 hours after injection. Additionally, narcotic medications used were converted to morphine equivalents based on the recommendations of the American Pain Society Pain Assessment and Management Initiative dosing guide available at https://globalrph.com/medical/narcotic/. Secondary endpoints comprised serum inflammatory markers preoperatively and at 48 and 72 hours postinjection, duration of surgery (hours), time to extubation after surgery (hours), intensive care unit (ICU) and hospital length of stay (LOS, days), and evaluation of postoperative nausea and vomiting (PONV).

### Assessment of serum inflammatory markers using enzyme-linked immunosorbent assay (ELISA)

The Human CRP (C-reactive protein) Instant ELISA Kit (BMS288INST) measures CRP levels in human serum with a sensitivity of 3.0 pg/mL and a detection range from 78 to 5000 pg/mL. The Human TNF-α (tumor necrosis factor-alpha) ELISA Kit (BMS2034) provides sensitive detection of TNF-α in human serum, with a sensitivity of 5.0 pg/mL and measures concentrations from 23 to 1500 pg/mL, showing low inter- and intra-batch variability (8.1% and 7.7%, respectively). The Human IL-1β (Interleukin-1beta) Instant ELISA Kit (BMS224INST) has a sensitivity of 0.7 pg/mL and measures IL-1β levels within a range of 7.8–500 pg/mL, with inter-y and intra-assay variabilities of 10.3% and 7.6%, respectively. The Human IL-6 ELISA Kit (EH2IL6) detects IL-6 with a sensitivity below 1 pg/mL and measures concentrations from 10.24 to 400 pg/mL, showing both inter- and intra-assay variabilities below 10%. These ELISA kits are available from Thermo Fisher Scientific Inc. (USA).

### Sample size calculation

The sample size calculation for this study was based on a previous study comparing postoperative pain scores at 24 hours between the Lip-BPVC and BPVC groups,^16)^ which yielded an effect size (d) of 0.548. Using G*Power software version 3.1.9.2 for a 2-sample t-test with equal group sizes and aiming for 80% power, a total of 84 participants (42 per group) were required. To account for potential 5% dropout rate, 41 participants per group were enrolled, ensuring an adequate sample size to meet the study's statistical power requirements.

### Statistical analysis

Statistical analysis was conducted using GraphPad Prism software. The normality of data distribution was assessed using the Shapiro–Wilk test. Non-normally distributed data are presented as median with interquartile range (IQR), and comparisons between groups were analyzed using the Mann–Whitney U-test. Normally distributed data are expressed as mean ± standard deviation (SD), with group comparisons assessed by the unpaired t-test. Categorical data were compared using the chi-square test. Statistical significance was defined as *P* <0.05.

## Results

### Demographic and clinical baseline characteristics

As shown in **Table 1**, a comparison was made between the Lip-BPVC group (n = 41) and the BPVC group (n = 41) across various demographic and clinical baseline characteristics. Both groups had a median age of 63 years and similar proportions of female patients, body mass index, and preoperative LVEF (all *P* >0.05). American Society of Anesthesiologists classifications and smoking status were also comparable (all *P* >0.05). The incidence of diabetes, hypertension, chronic lung disease, cerebrovascular disease, and history of myocardial infarction did not differ significantly between the groups (all *P* >0.05), indicating a well-matched cohort for comparison.

**Table 1:** Demographic and clinical baseline characteristics

Characteristics	Lip-BPVC group (n = 41)	BPVC group (n = 41)	*P*
Age (years)	63 (57.7–68)	63 (58.4–68.1)	0.176
Sex			
Female	11	13	
Male	30	28	0.809
BMI (kg/m²)	25.26 (23.51–27.69)	24.91 (23.01–27.34)	0.071
Preoperative LVEF (%)	60 (58–61)	60 (57–61)	0.164
ASA classification			
2	1	0	
3	9	12	
4	31	29	0.474
Smoking status			
Current	0	2	
Former	18	15	
Never	23	24	0.318
Diabetes	13	15	0.816
Hypertension	26	24	0.821
Chronic lung disease	1	1	1.000
Cerebrovascular disease	4	6	0.738
History of myocardial infarction	13	10	0.624

Lip-BPVC: liposomal bupivacaine; BPVC: standard bupivacaine; BMI: body mass index; LVEF: left ventricular ejection fraction; ASA: American Society of Anesthesiologists

### Pain scores measured at postinjection time points

**Fig. 2:**
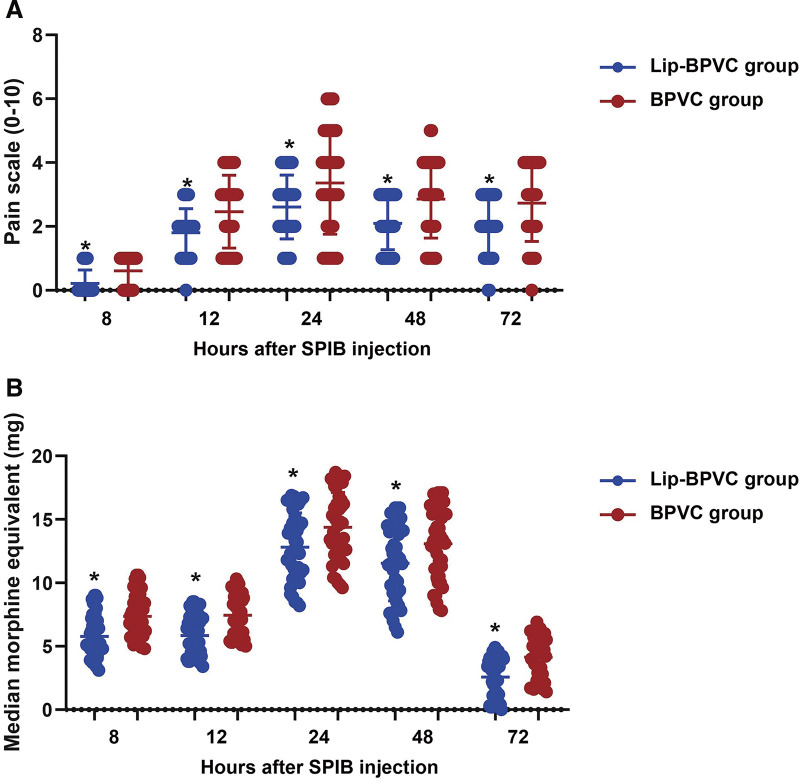
Pain scores and morphine equivalents postinjection. (**A**) Pain scores ranging from 0 to 10, assessed using the nonverbal pain scale or VAS at 8, 12, 24, 48, and 72 hours after injection. (**B**) Pain medication usage was converted to OME at 8, 12, 24, 48, and 72 hours after injection. An asterisk (*) indicates *P* <0.05 compared to the BPVC group. Lip-BPVC: liposomal bupivacaine; SPIB: superficial parasternal intercostal plane block; VAS: visual analog scale; OME: oral morphine equivalents.

The Lip-BPVC group demonstrated lower pain scores compared to the BPVC group throughout the entire 72-hour postinjection period (all *P* <0.05, **Fig. 2A**). At 8 hours postinjection, the Lip-BPVC group had a median pain score of 0 (0–0), significantly lower than the BPVC group's median pain score of 1 (0–1). This trend continued at 12 hours, with the Lip-BPVC group reporting a median pain score of 2 (1–2) compared to 3 (1–3) in the BPVC group. At 24 hours, the pain scores were 3 (2–3) for the Lip-BPVC group and 3 (2–5) for the BPVC group. At 48 hours, the Lip-BPVC group had a median pain score of 2 (1–3) compared to 3 (2–4) for the BPVC group. Finally, at 72 hours, the Lip-BPVC group reported a median pain score of 2 (1–3), while the BPVC group had a median pain score of 3 (2–4). These results indicate that the Lip-BPVC group consistently experienced less pain across all measured time points.

### Morphine equivalents measured at postinjection time points

In terms of morphine equivalents, the Lip-BPVC group consistently required fewer opioids (**Fig. 2B**). At 8 hours, the median morphine equivalent (mg) for the Lip-BPVC group was 5.4 (4.6–7.1) compared to 7.20 (6.00–8.55) for the BPVC group (*P* <0.001). This difference persisted at 12 hours (5.60 [4.50–7.20] vs. 7.50 [5.90–8.85]), 24 hours (12.50 [10.55–15.00] vs. 14.50 [12.20–16.65]), 48 hours (11.70 [9.05–14.10] vs. 13.40 [10.45–15.50]), and 72 hours (3.20 [0.85–4.00] vs. 4.70 [2.40–5.55], all *P* <0.05). Total postinjection 72-hour morphine equivalents (mg) were 38.53 ± 5.14 in the Lip-BPVC group and 46.40 ± 5.07 in the BPVC group (*P* <0.001). Overall, these results show that the Lip-BPVC group had a significantly lower requirement for opioid analgesics, indicating a more effective pain management regimen.

### Inflammatory response comparison between Lip-BPVC and BPVC groups postsurgery

As shown in **Table 2**, baseline serum levels of CRP, TNF-α, IL-6, and IL-1β were comparable between the Lip-BPVC and BPVC groups preoperatively. However, at 24 hours postinjection, significantly higher levels of CRP, TNF-α, IL-6, and IL-1β were observed in the Lip-BPVC group compared to the BPVC group (all *P* <0.05), indicating a more pronounced inflammatory response. This difference persisted at 48 hours postinjection, highlighting sustained disparities in inflammatory biomarker levels between the 2 groups (all *P* <0.05). No significant differences in serum levels of CRP, TNF-α, IL-6, and IL-1β were found at 72 hours postinjection between the Lip-BPVC and BPVC groups (all *P* >0.05).

**Table 2:** Assessment of serum inflammatory markers in Lip-BPVC and standard BPVC groups before and 24–72 hours after injection

	Lip-BPVC group (n = 41)	BPVC group (n = 41)	*P*
**CRP (pg/mL)**			
Preoperative	1295.0 ± 640.1	1288.0 ± 697.9	0.965
24 hours postinjection	2741.0 ± 778.6	2022.0 ± 706.9	<0.001
48 hours postinjection	1992.0 ± 730.5	1654.0 ± 753.0	0.043
72 hours postinjection	1643.0 ± 697.0	1458.0 ± 744.3	0.249
**TNF-α (pg/mL)**			
Preoperative	445.1 ± 225.4	397.3 ± 204.3	0.318
24 hours postinjection	610.7 ± 236.5	493.3 ± 211.5	0.02
48 hours postinjection	527.6 ± 218.9	429.6 ± 208.9	0.041
72 hours postinjection	487.2 ± 219.9	464.6 ± 190.4	0.62
**IL-6 (pg/mL)**			
Preoperative	116.8 ± 40.06	122.9 ± 48.13	0.533
24 hours postinjection	255.8 ± 47.38	192.2 ± 53.82	<0.001
48 hours postinjection	187.4 ± 54.49	154.9 ± 55.58	0.009
72 hours postinjection	152.8 ± 45.03	137.4 ± 51.77	0.153
**IL-1β (pg/mL)**			
Preoperative	104.2 ± 57.61	106.5 ± 54.58	0.851
24 hours postinjection	241.0 ± 67.77	178.6 ± 55.85	<0.001
48 hours postinjection	170.0 ± 80.71	133.3 ± 60.08	0.022
72 hours postinjection	135.3 ± 66.41	128.6 ± 60.17	0.637

CRP: C-reactive protein; TNF-α: tumor necrosis factor-alpha; IL-6: interleukin-6; IL-1β: interleukin-1beta. Lip-BPVC: liposomal bupivacaine; BPVC: bupivacaine

### Comparison of surgical and recovery outcomes between BPVC and Lip-BPVC groups

**Fig. 3:**
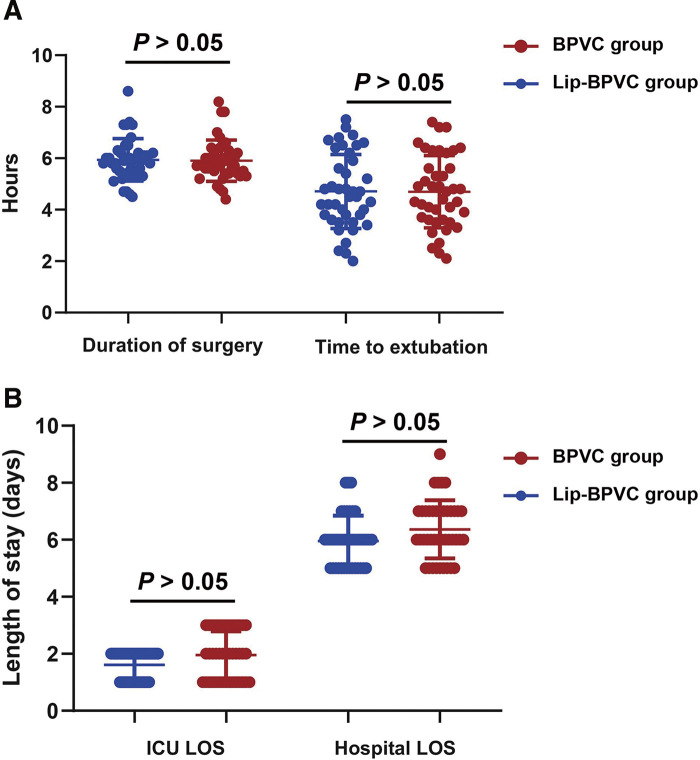
Comparison of surgical and recovery outcomes between BPVC and Lip-BPVC groups. (**A**) Duration of surgery (hours) and time to extubation after surgery (hours). (**B**) Intensive care unit (ICU and hospital LOS. Lip-BPVC: liposomal bupivacaine; LOS: length of stay; ICU: intensive care unit.

As shown in **Fig. 3**, the duration of surgery was comparable between the BPVC and Lip-BPVC groups, with median times of 5.7 hours (IQR 5.4–6.25) and 5.9 hours (IQR 5.4–6.2), respectively (*P* = 0.738). Similarly, the median time to extubation postsurgery showed no significant difference between groups, with 4.7 hours (IQR 3.6–5.9) for BPVC and 4.5 hours (IQR 3.7–6.05) for Lip-BPVC (*P* = 0.963). Regarding ICU length of stay, no statistically significant difference was observed: the BPVC group had a median 2 days (IQR 1–3) versus a median of 2 days (IQR 1–2) for the Lip-BPVC group (*P* = 0.071). The hospital length of stay also showed no significant difference, with a median of 6 days (IQR 5–6) in the Lip-BPVC group compared to a median of 6 days (IQR 6–7) in the BPVC group (*P* = 0.054). The incidence of PONV within the first 24 hours after surgery was comparable between groups, with rates of 24.39% in the Lip-BPVC group and 29.27% in the BPVC group (*P* = 0.804).

## Discussion

The results of this randomized controlled trial comparing Lip-BPVC and standard BPVC in SPIB for patients undergoing elective CABG via median sternotomy reveal significant findings. Lip-BPVC was associated with markedly lower postoperative pain scores and reduced opioid use, alongside a favorable inflammatory response profile. These findings have crucial implications for enhancing postoperative pain management and recovery in cardiac surgery patients.

While previous research has found no significant advantages of Lip-BPVC over conventional formulations in various surgical contexts, such as primary total hip arthroplasty,^17)^ midurethral sling placement,^18)^ and transversus abdominis plane blocks after abdominally based microvascular breast reconstruction,^19)^ our study demonstrates superior pain relief with Lip-BPVC across all assessed postoperative time points. The selection of the 72-hour timepoint corresponds to the claimed pharmacological duration of the slow-release formulation.^16)^ The consistently lower pain scores in the Lip-BPVC group at 8, 12, 24, 48, and 72 hours postinjection indicate sustained analgesic effects, likely attributed to Lip-BPVC’s extended-release properties. This prolonged pain relief translated into reduced opioid requirements, as evidenced by consistently lower morphine equivalents in the Lip-BPVC group. These findings are consistent with prior studies showing Lip-BPVC’s efficacy in prolonging pain control and reducing opioid consumption in various surgical settings. For example, a study evaluating Lip-BPVC in supraclavicular brachial plexus blocks for distal radial fracture fixation found significant reductions in postoperative pain during the initial 48 hours compared to standard BPVC alone.^20)^ Jacobus et al. compared Lip-BPVC with conventional BPVC in exploratory lingual nerve microsurgery, revealing lower postoperative pain and opioid use with Lip-BPVC.^21)^ However, a randomized controlled trial comparing Lip-BPVC with standard BPVC and lidocaine during midurethral sling placement showed no significant difference in reducing postoperative day 1 pain scores or overall analgesic consumption, despite slightly improved early quality of recovery scores with Lip-BPVC.^18)^

In our study, Lip-BPVC demonstrated an initial heightened inflammatory response postoperatively compared to standard BPVC, as indicated by significantly lower levels of CRP, TNF-α, IL-6, and IL-1β at 24 and 48 hours postinjection with BPVC. However, by 72 hours, inflammatory markers did not differ significantly between Lip-BPVC and BPVC groups, suggesting a transient effect. Previous research on horses showed that subcutaneous perineural injection of Lip-BPVC for abaxial sesamoid nerve blocks provided comparable pain relief to standard BPVC, despite mild inflammation noted in 30% of Lip-BPVC injection sites versus none with BPVC alone.^22)^ Similarly, McAlvin et al. observed that, compared to standard BPVC solutions, Lip-BPVC (Exparel) caused slightly higher tissue inflammation scores immediately postinjection, although long-term inflammation after 2 weeks was comparable to that seen with low-concentration BPVC HCl, with no observed neurotoxic effects.^23)^ Moreover, a study by Ferré et al. found that while both Lip-BPVC and BPVC caused mild neural inflammation in mice receiving perineural sciatic nerve injections, Lip-BPVC led to more frequent inflammation.^24)^ We hypothesized that the sustained drug release and prolonged local exposure associated with Lip-BPVC’s lipid-based delivery system may contribute to the higher inflammatory response observed, as prolonged exposure can enhance inflammation.^23–25)^

Despite significant differences in pain scores and inflammatory markers, variables such as surgical duration, time to extubation, ICU length of stay, and total hospital stay did not differ significantly between the 2 groups. These findings suggest that while Lip-BPVC provided effective pain relief, the sustained release and prolonged local exposure associated with Lip-BPVC could contribute to a heightened inflammatory response, potentially affecting postoperative recovery, particularly with respect to hospital stay and complications related to inflammation. However, the improved pain management and reduced opioid requirements with Lip-BPVC remain clinically significant outcomes enhancing patient comfort and satisfaction. Given that opioid use is a known risk factor for PONV, the reduced opioid consumption in the Lip-BPVC group might have been expected to lower PONV rates. The similar incidence of PONV in both groups could be attributed to other factors influencing nausea and vomiting, such as individual patient characteristics and the use of prophylactic antiemetics.

However, it is important to acknowledge some limitations. Our assessment of inflammation was confined to a short-term evaluation period and a limited sample size, which may not fully capture extended inflammatory responses or rare adverse events that could impact clinical outcomes. Additionally, we did not extensively assess postoperative complications, such as pleural or pericardial effusions and dehydration issues, which may be influenced by the inflammatory response and could potentially affect recovery and hospital stay. Further studies with larger sample sizes and longer follow-up periods are needed to fully evaluate the long-term impact of Lip-BPVC on both inflammation and postoperative complications.

## Conclusion

In summary, this study demonstrates that Lip-BPVC provides superior pain relief and reduces opioid use compared to standard BPVC in patients undergoing CABG via median sternotomy. Despite initial concerns about a heightened inflammatory response with Lip-BPVC, inflammatory markers normalized by 72 hours postinjection. These findings highlight Lip-BPVC's potential to enhance postoperative pain management without compromising safety or recovery outcomes in cardiac surgery patients.

## Declarations

### Consent for publication

We have obtained the patient’s consent for publication.

### Ethics approval and consent to participate

The study protocols involving human participants were approved by the Ethics Committee of the Quzhou Affiliated Hospital of Wenzhou Medical University, Quzhou People’ s Hospital, and written informed consent was obtained from all participants.

### Availability of data and materials

The datasets generated during and/or analyzed during the current study are available from the corresponding author upon reasonable request.

### Funding

This research was supported by the 2023 Clinical Research Project of Analgesia Action of the Medical Empowerment Public Welfare Special Fund of the Red Cross Foundation of China under Grant No. 2023-925.

### Authors Contributions

Rong-En Qiu: conceptualization (lead); methodology (lead); investigation (lead); writing—original draft (equal); writing—review and editing (lead).

Yun-Ping Lan: supervision (lead); project administration (lead); funding acquisition (lead); writing—review and editing (equal).

Shan Liu: investigation (equal); data curation (equal); visualization (equal); writing—review and editing (equal).

Xiang-Yu Fang: conceptualization (supporting); methodology (supporting); resources (supporting); writing—review and editing (equal).

Yun-Feng Zhang: revision (lead); visualization (equal); writing—review and editing (equal). All authors have read and approved the manuscript.

### Disclosure statement

The authors declare that they have no competing interests.
